# Winter home range and habitat selection differs among breeding populations of herring gulls in eastern North America

**DOI:** 10.1186/s40462-019-0152-x

**Published:** 2019-03-07

**Authors:** Christine M. Anderson, H. Grant Gilchrist, Robert A. Ronconi, Katherine R. Shlepr, Daniel E. Clark, D. V. Chip Weseloh, Gregory J. Roberston, Mark L. Mallory

**Affiliations:** 1 Department of Biology, Acadia University, 33 Westwood Ave, Wolfville, NS B4P 2R6 Canada; 2 Wildlife Research Division, Environment and Climate Change Canada, National Wildlife Research Centre, Ottawa, ON K1S 5B6 Canada; 3 Canadian Wildlife Service, Environment and Climate Change Canada, 45 Alderney Dr, Dartmouth, NS B2Y 2N6 Canada; 4 Atlantic Lab for Avian Research, Department of Biology, University of New Brunswick, P.O. Box 4400, 10 Bailey Drive, Fredericton, NB E3B 5A3 Canada; 5 Massachusetts Department of Conservation and Recreation, Division of Water Supply Protection, 485 Ware Road, Belchertown, MA 01007 USA; 6 Canadian Wildlife Service, Environment and Climate Change Canada, 4905 Dufferin Ave, Toronto, ON M3H 5T4 Canada; 7 Wildlife Research Division, Environment and Climate Change Canada, 6 Bruce Street, Mount Pearl, NL A1N 4T3 Canada

**Keywords:** Bird, Non-breeding, Home range, Habitat use, Tracking

## Abstract

**Background:**

Recognizing the factors influencing migratory individuals throughout their annual cycle is important for understanding the drivers of population dynamics. Previous studies have found that Herring Gulls (*Larus argentatus*) in the Atlantic region have lower survival rates than those in the Great Lakes and the Arctic. One possible explanation for divergent survival rates among these populations is differences in their non-breeding habitats.

**Methods:**

We tracked Herring Gulls from five populations, breeding in the eastern Arctic, the Great Lakes, Newfoundland, Sable Island, and the Bay of Fundy. We assessed the extent of migratory connectivity between breeding and wintering sites, and tested if there were differences in home range size or habitat selection among these populations during the winter.

**Results:**

The tracked Herring Gulls had strong migratory connectivity between their breeding and wintering areas. We found that Herring Gulls from the Arctic spent most of the winter in marine habitats, while the other populations used a wider variety of habitats. However, the Newfoundland and Sable Island populations selected for urban habitats, and almost all individuals the specialized in urban habitats came from one of the three Atlantic populations.

**Conclusions:**

Our results suggest that there could potentially be a link between urban habitat use during the winter and reduced adult survival in Atlantic Canada Herring Gulls.

**Electronic supplementary material:**

The online version of this article (10.1186/s40462-019-0152-x) contains supplementary material, which is available to authorized users.

## Introduction

Linking the spatially discrete parts of the annual cycle for migratory birds is useful for understanding how population dynamics are shaped by events throughout the entire year [1]. The geographic structure of a migratory network can have a significant influence on population dynamics [2–4]. In populations with strong connectivity, individuals from one breeding population move to the same location to form one non-breeding population, while in populations with weak connectivity, individuals from a breeding population move to a variety of locations, and non-breeding populations are composed of individuals from multiple breeding populations [5]. Consequently, in populations with strong migratory connectivity, negative environmental impacts on birds in wintering areas should be observed in breeding populations as well [6]. Habitat loss and degradation in wintering areas has been identified as a major cause of bird population declines, and conservation actions are more effective when migratory connectivity is considered [7].

Survival rates vary among Herring Gull (*Larus argentatus*) populations in eastern North America, and the reason why is not clear. Estimates of apparent adult survival in the Atlantic region have ranged from 0.80 to 0.83 [8–10], while estimates from the Arctic and the Great Lakes were considerably higher at 0.87 and 0.91, respectively [11, 12]. Although these differences in survival rates appear small, they translate to substantial differences in life expectancy and number of breeding seasons. While an average adult will have 19 breeding seasons at 95% survival, the number of breeding seasons is reduced to 10 at 90% survival, and six at 85% survival [13]. Declines in Herring Gull abundance have been observed at colonies across Atlantic Canada and the northeastern United States [14, 15]. These declines have been correlated with decreases in fisheries discards due to the collapse of groundfish fisheries and reduced forage fish availability [16, 17], however this is most likely to influence abundance through effects on chick survival rather than adult survival [18, 19].

Robertson et al. [10] speculated that lower survival rates in the Atlantic Canada population may be due, in part, to differences among the wintering home ranges of these populations. Adult Herring Gulls from Atlantic Canada are most often resighted in New England and New York [20], a heavily populated and industrialized region [21]. Gulls are actively managed in this region, through shooting and hazing at airports, landfills, and industrial buildings where gulls nest on roofs [22]. Moreover, survival rates could also be linked to food availability, trophic niche and food quality/contamination within certain habitats [23].

It is not clear how Herring Gulls budget their time between different habitats during the winter, and how winter habitat use differs between individuals and populations. Much of the research on Herring Gull habitat use in North America has been conducted during the breeding season [17, 24–26], but less is known about their winter habitat preferences. During the winter non-breeding period, Herring Gulls are observed in a wide variety of habitats, including coastal areas, the continental shelves, the lower Great Lakes, and major rivers [27]. Herring Gulls are also abundant in urban areas and human-dominated habitats, resulting in regular conflicts with people, leading to management efforts to reduce their abundance [22].

In this study, we analyzed the wintering distribution of Herring Gull populations breeding in the eastern Arctic, the Great Lakes, Newfoundland, Sable Island, and the Bay of Fundy to explore the following questions: (1) what are their winter home ranges, (2) what is the degree of migratory connectivity, and (3) are there differences in habitat use and selection among these populations during the winter? Given that survival rates in the Atlantic region are lower than the Arctic and Great Lakes populations, we predicted Herring Gulls breeding in Newfoundland, Sable Island, and the Bay of Fundy would use anthropogenic habitats more frequently than the other populations, as these habitats likely incur higher risk of mortality.

## Methods

### Tracking

**Fig. 1:**
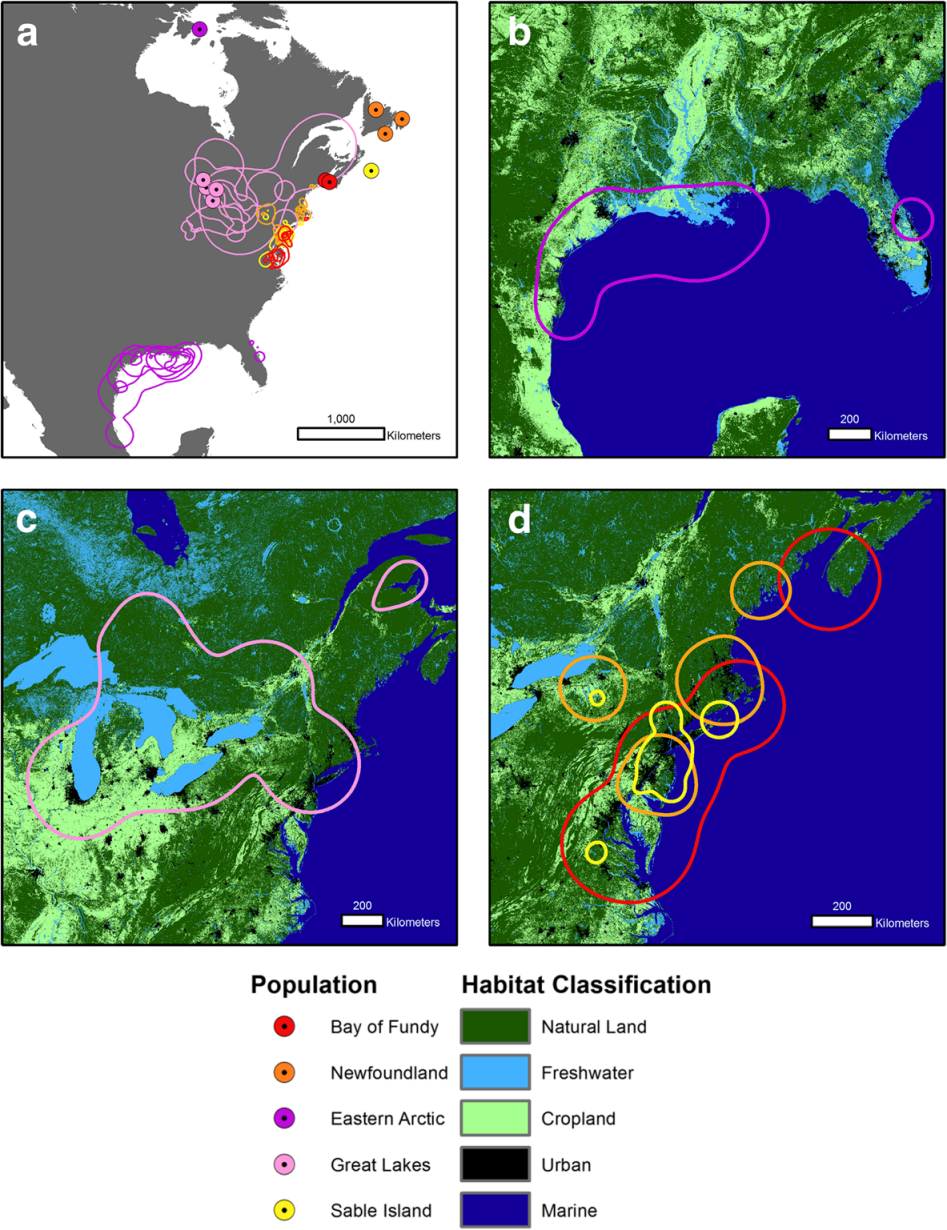
**a** Breeding locations (circles) and individual 90% kernel utilization distributions for each bird Herring Gull tracked from the eastern Arctic, the Great Lakes, Newfoundland, Sable Island, and the Bay of Fundy, illustrating the extent of migratory connectivity between populations. **b**-**d** Habitat availability within the population 90% kernel utilization distributions for the **b** eastern Arctic, **c** Great Lakes, and **d** Newfoundland, Sable Island, and the Bay of Fundy populations

We used tracking data from five populations of Herring Gulls, breeding in the eastern Canadian Arctic, the Great Lakes, Newfoundland, Sable Island (offshore from Nova Scotia), and the Bay of Fundy, with observations between 1999 and 2016 (Table 1, Fig. 1a). Tracking devices were deployed at one site in the eastern Arctic (Southampton Island, NU, 64.01^0^ N, 81.75^0^ W), two sites in the Great Lakes (Agawa Rocks, Lake Superior, 47.33^0^ N, 84.68^0^ W; and Double Island, Lake Huron 45.55^0^ N, 80.50^0^ W), one site in Newfoundland (Witless Bay, 47.26^0^ N, 52.77^0^ W), on Sable Island (43.92^0^ N, 60.00^0^ W), at two sites in the Bay of Fundy (Kent Island, NB, 44.57^0^ N, 66.75^0^ W; Brier Island, NS 44.25^0^ N, 66.33^0^ W), as well as within 75 km of the Quabbin Reservoir, MA (42.40^0^ N, 72.31^0^ W). Devices were deployed during the incubation period at breeding locations, except for the devices deployed in Massachusetts, which were deployed during the winter [28]. These birds were subsequently tracked to their breeding locations in Newfoundland (*n* = 3) and the Great Lakes *(n* = 1), and assigned to the appropriate breeding population in our analyses.

**Table 1:** Details of tracking device deployment for collecting Herring Gull movement data used in this study

Breeding Population	Deployment Location	Years of Tracking	Type of Device	*n*	Size	Duty Cycle	Attachment Method	Capture Method
Eastern Arctic	Southampton Island, Nunavut	2008, 2013–2015	Doppler PTT	8	18 g	10 h on, 24 h off	Leg loop harness [28]	Drop Trap [29]
Newfoundland	Witless Bay, Within 75 km of Quabbin Reservoir, Massachusetts; Witless Bay, NL	2009–2013 2015–2016	Doppler PTT, GPS PTT	9	11-30 g	8 h on, 18 h off; 6 locations per day	Chest harness [31] Leg loop harness	Net Launcher [28] Bow-net [29]
Great Lakes	Double Island, Lake Huron; Agawa Rocks, Lake Superior; Within 75 km of Quabbin Reservoir, Massachusetts	1999–2001, 2008–2009	Doppler PTT	8	20-30 g	8 h on, 72 h off	Chest harness	Drop Trap
Sable Island	Sable Island, NS	2012–2016	GPS PTT	8	22 g	8 locations per day	Leg loop harness	Bow-net
Bay of Fundy	Kent Island, NB; Brier Island, NS	2009–2010, 2014–2015	Doppler PTT, GPS logger	10	17-18 g	6 locations per day	Leg loop harness	Drop Trap

Breeding birds were captured using a self-triggering wire mesh drop trap over their nest [29]. Wintering birds caught in Massachusetts were captured using a Coda net launcher hidden under a pickup truck. Bait was placed in front of the net, and the launcher was detonated from inside the truck’s cab [30]. At the Massachusetts and Great Lakes sites, devices were attached using variations of a chest harness, with the transmitter resting on the upper back, secured with loops around the wings and joined at the chest [31]. At the eastern Arctic, Newfoundland, Sable Island, and Bay of Fundy sites, devices were attached using a leg loop harness, with the transmitter resting on the lower back and secured with loops around the bird’s legs [32]. These harnesses have not been observed to influence survival or behaviour of large gulls [33].

Birds were equipped either with Ecotone devices, which archive global positioning system (GPS) data internally and transmit data to a base station at the breeding site; or with platform terminal transmitters (PTTs), which derive location data from either GPS and/or Doppler shifts and transmit through the Argos satellite system [34]. Doppler-derived data were collated and processed by Argos, and categorized into four location error classes [34]. Data from GPS were considered to have a fixed location with an error radius of 0 m. Tracking devices weighed 11.5 g to 30 g (< 3% of average Herring Gull body mass) and were programmed with a variety of duty cycles (Table 1). Tracking devices do not appear to alter the behaviour, survival, and reproductive success of large gulls [33, 35, 36]. After removing tracks that did not contain both breeding and wintering locations, we obtained tracks from 43 individuals, with eight to ten individuals from each population (Table 1). Most individuals were tracked for 1 year, but 14 birds were tracked for 2–5 years, giving a total of 65 winter tracks.

### Preparation of movement data

We used Bayesian State–Space Models to model the birds’ movement paths from location data, which both accounts for differences in location errors from the different tracking methods and provides location estimates at regular 24 h intervals (See Additional file 1 for a full description). To delineate the wintering period, we visualized the tracks to identify the dates where autumn migration ceased and spring migration started, which can be observed as a sudden change the distance travelled per day. Herring Gulls from the Great Lakes are a resident population; there are some intra-seasonal differences their movement and use of space, but they do not undertake a clear migration to a distinct wintering area [37, 38]. For the Great Lakes population, we defined the wintering period as starting when the individual travelled away from their nest site, and ending when they returned. We confirmed that our definitions of the wintering period for each population seemed appropriate, as there were no major variations in habitat use or the location of home ranges when the winter was subdivided into several smaller periods. The timing and length of the wintering period varied widely among individuals and populations (Table 2). We excluded annual wintering periods with less than 30 days of tracking data.

**Table 2:** Duration (*n* = 2, 5, 14, 13, 12), arrival dates (*n* = 8, 9, 12, 18, 13), and departure dates (*n* = 2, 5, 14, 13, 12) for the wintering period of Herring Gulls tracked from the eastern Arctic, the Great Lakes, Newfoundland, Sable Island, and the Bay of Fundy. Most individuals were tracked for 1 year, but some were tracked for up to 5 years. All summary statistics are presented as mean ± SD (range)

Breeding Population	Winter Duration		Arrival Date		Departure Date	
Eastern Arctic	144	±70	Nov 28	±32	May 5	±18
	(94–193)		(Oct 29 - Jan 19)		(Apr 22 – May 18)	
Great Lakes	164	±66	Aug 18	±34	Mar 2	±28
	(89–241)		(Jul 30 - Nov 13)		(Feb 9 - Apr 3)	
Newfoundland	143	±41	Oct 31	±28	Mar 30	±9
	(33–202)		(Sep 27 - Dec 21)		(Mar 16 - Apr 16)	
Sable Island	176	±53	Sept 29	±58	Mar 25	±8
	(89–253)		(July 7 - Jan 18)		(Mar 15 - Apr 12)	
Bay of Fundy	162	±75	Oct 16	±64	Apr 2	±21
	(79–271)		(July 13 - Dec 28)		(Mar 8 - May 14)	

### Winter home range

We calculated the extent and area of home range for individual birds and for each population as 90% kernel density utilization distributions (kernel UD) using the R package *adehabitatHR* [38]. Given the small sample sizes of tracked individuals from each population, we calculated the population home range area for a sequence of sample sizes from 1 to *n* for each population and plotted to see if the area approached an asymptote (as in [39]). The Newfoundland, Sable Island and Bay of Fundy populations did approach an asymptote, while the eastern Arctic and Great Lakes populations still showed some variation in the rate of change at the maximum sample size, suggesting that the home range for these two populations may be underestimated (Additional file 1: Figure S1).

We assessed the strength of migratory connectivity between breeding and non-breeding sites by using a Mantel correlation coefficient [40], calculated with the R package *ade4* [41]. We defined breeding sites as an individual’s nesting colony location, and wintering sites as the centroid of an individual’s winter home range. We used the Geographic Distance Matrix Generator v. 1.2.3 [42] to create two pairwise matrices of the distance between all individuals, one for each season. We tested for a correlation between the two matrices, with 9999 permutations to determine statistical significance. A significant positive correlation suggests strong migratory connectivity.

### Winter habitat use and selection

We obtained habitat data from the 2010 North American Land Cover database [43]. In this database, remote sensing data from Moderate Resolution Imaging Spectroradiometer [44] have been categorized into 19 land cover classes at a 250 m spatial resolution. We used ArcGIS to simplify the dataset into five land cover classes: marine, freshwater, cropland, urban, and natural. The two aquatic habitats, “Marine” and “Freshwater”, were both originally classified as water covering at least 75% of a 250 m pixel. We separated the two using the ocean coastline feature from the 2010 North America Environmental Atlas Bathymetry dataset [45]. The “Wetland” cover class consisted of areas dominated by wetland vegetation where water is present for a substantial period annually, and was merged with the “Freshwater” class. We retained two anthropogenic land cover classes from the original dataset classifications: “Urban” was defined as areas containing ≥30% urban constructed materials for human activities such as buildings and roads, and “Cropland” was defined as areas dominated by intensively managed crops, with crop vegetation accounting for > 20% of total vegetation. The remaining 16 land classes were grouped as a single “Natural Land” land cover class, which included forests, shrublands, grasslands, and barren lands.

To assess habitat use, we extracted the habitat type associated with each observed location within the population kernel UD. We used logistic resource selection functions (RSFs) with a use-availability design to assess habitat selection for each population. We defined habitat availability at the level of the population home range (Design II approach [46]), within which we assessed individual habitat use (third-order selection [47]). We randomly sampled available locations within the population kernel UD at a 10:1 ratio to used locations [48]. To account for differences in tracking length and behaviour, we included individual as a random effect in our models [49]. With this design, RSFs are interpreted as a ranking of which habitats are most strongly correlated with use rather than a probability of use [50]. We checked goodness-of-fit using a likelihood ratio test with a null model.

To summarize how individuals budgeted their time between the five habitat types, we used principal components analysis (PCA) with Aitchison compositional scaling to account for proportional data [51]. We used ANOVA to assess if the first two principal components differed by population.

All maps are displayed using a North America Lambert Conformal Conic projection. Statistical analyses were performed in R version 3 [52]. Means are reported throughout the text ± standard deviation (SD). Measurements to determine sex were not available.

## Results

### Winter home range

Each breeding populations was tracked to a distinct winter home range, suggesting relatively strong migratory connectivity (Fig. 1a; Mantel correlation coefficient = 0.30, *p* = 0.001, *n* = 43) suggesting relatively strong migratory connectivity. Herring Gulls that bred on Southampton Island, NU spent most the winter offshore from Louisiana, Texas and Mexico. Herring Gulls that bred in the Great Lakes spent the majority of the winter in the Great Lakes basin, although individual birds spent time in Massachusetts, the Ottawa Valley in Ontario, and the Gaspé Peninsula in Quebec. The three populations from Atlantic Canada wintered in the northeastern United States: birds from Newfoundland ranging from Cape Cod to New Jersey birds from Sable Island concentrated around New Jersey, and birds from the Bay of Fundy ranging from New Jersey to Delaware Bay. Two individuals from the Bay of Fundy did not migrate, spending the entire winter in Nova Scotia within 50 km of their breeding site. For individuals that were tracked over the entire season, the wintering period was 160 (±57) days on average (Table 2).

The Great Lakes population had a winter home range area of 1,153,000 km^2^, larger than the eastern Arctic population (506,000 km^2^), the Bay of Fundy population (381,000 km^2^), and the Newfoundland population (181,000 km^2^), with the smallest being the Sable Island population (58,000 km^2^). Individual home range size had a strong left skew: 34 individuals had home range of 100 km^2^ to 90,00 km^2^, while nine individuals had home ranges of 170,000 km^2^ to 1,446,000 km^2^ (overall median = 16,000 km^2^). Individual home range sizes did not differ by population (*F* = 1.15, *p* = 0.35).

### Winter habitat use and selection

**Fig. 2:**
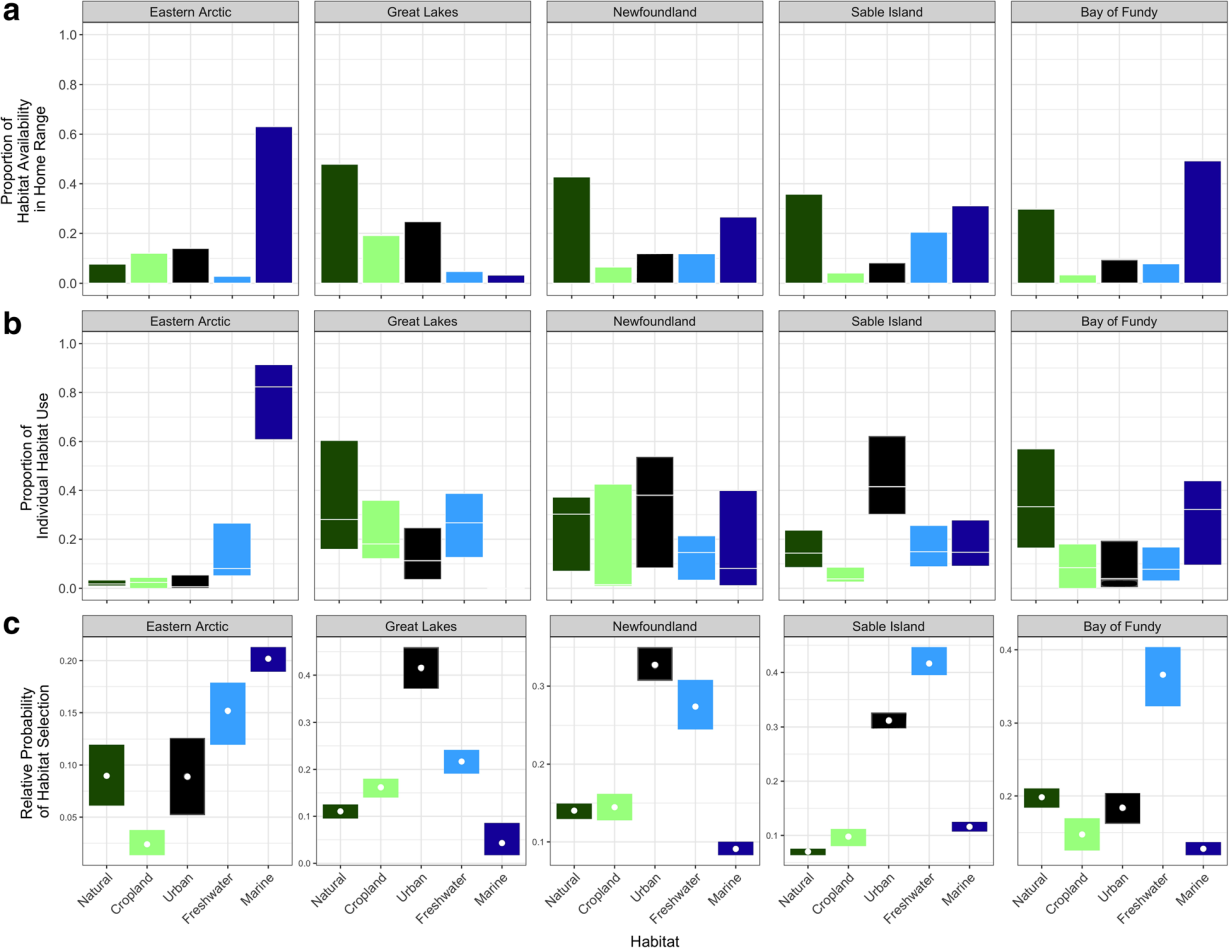
**a** Proportion of marine, natural land, cropland, urban, and freshwater habitats available within the winter home range of Herring Gull populations from the eastern Arctic, the Great Lakes, Newfoundland, Sable Island, and the Bay of Fundy; **b** Proportion of time spent in each habitat by individual Herring Gulls. Boxes represent the 95% confidence interval of the median individual habitat use for each population, acquired through 1000-fold bootstrapping; **c** Relative probability of habitat selection predicted by logistic resource selection functions

Randomized available habitat in the winter home range of Arctic-breeding Herring Gulls was dominated by marine habitat (63%; Fig. 2b). For the Great Lakes population, available habitat was largely composed of natural (48%) and cropland (25%) habitats (Fig. 2c). For the Newfoundland, Sable Island, and Bay of Fundy populations (Fig. 2d), available locations were comprised of natural (42, 37, and 30% respectively) and marine (26, 30, and 50%) habitats, as well as a higher proportion of available urban habitat (20%) in the Sable Island home range.

Individuals from the eastern Arctic spent most of their time in marine habitats (82%). They appeared to use both coastal and pelagic habitats; locations in marine habitats were a mean of 43 km offshore, up to a maximum of 286 km. Birds from the three Atlantic populations largely used marine habitats close to shore, with a mean distance of 8 km from the coast. Herring Gulls from the Great Lakes used natural (30%), freshwater (27%), cropland (24%) and urban (17%) habitats fairly equally. The three Atlantic populations spent most of their time in a combination of natural (Newfoundland and Bay of Fundy), urban (Newfoundland and Sable Island) or marine habitats (Bay of Fundy and Sable Island).

Resource selection functions showed that relative to availability, all five selected for freshwater habitats (Table 3). The eastern Arctic population also used marine habitats in greater proportion than their availability. The Great Lakes, Newfoundland, and Sable Island populations showed selective use of urban habitats (Fig. 2c).

**Table 3:** Model coefficient β, coefficient standard error (SE *β),* and Log Odds Ratio for habitat selection of wintering Herring Gulls breeding in the eastern Arctic, the Great Lakes, Newfoundland, Sable Island, and the Bay of Fundy

Population	*Habitat*	*β*	SE *β*	Log Odds Ratio
Eastern Arctic	Intercept (Urban)	−2.33	0.30	–
	Natural Land	0.01	0.35	1.01
	Cropland	−1.38	0.32	0.25
	Freshwater	0.61	0.40	1.84
	Marine	0.95	0.31	2.60
Great Lakes	Intercept (Urban)	−0.34	0.12	–
	Natural Land	−1.74	0.14	0.17
	Cropland	−1.30	0.15	0.27
	Freshwater	−0.94	0.15	0.39
	Marine	−2.76	0.47	0.06
Newfoundland	Intercept (Urban)	−0.72	0.05	–
	Natural Land	−1.09	0.07	0.34
	Cropland	−1.06	0.09	0.35
	Freshwater	−0.26	0.09	0.77
	Marine	−1.58	0.08	0.21
Sable Island	Intercept (Urban)	−0.79	0.03	–
	Natural Land	−1.79	0.06	0.17
	Cropland	−1.43	0.10	0.24
	Freshwater	0.45	0.07	1.57
	Marine	−1.24	0.06	0.29
Bay of Fundy	Intercept (Urban)	−1.49	0.08	–
	Natural Land	0.09	0.10	1.10
	Cropland	−0.27	0.12	0.77
	Freshwater	0.94	0.13	2.56
	Marine	−0.43	0.10	0.65

### Principal components analysis

**Fig. 3:**
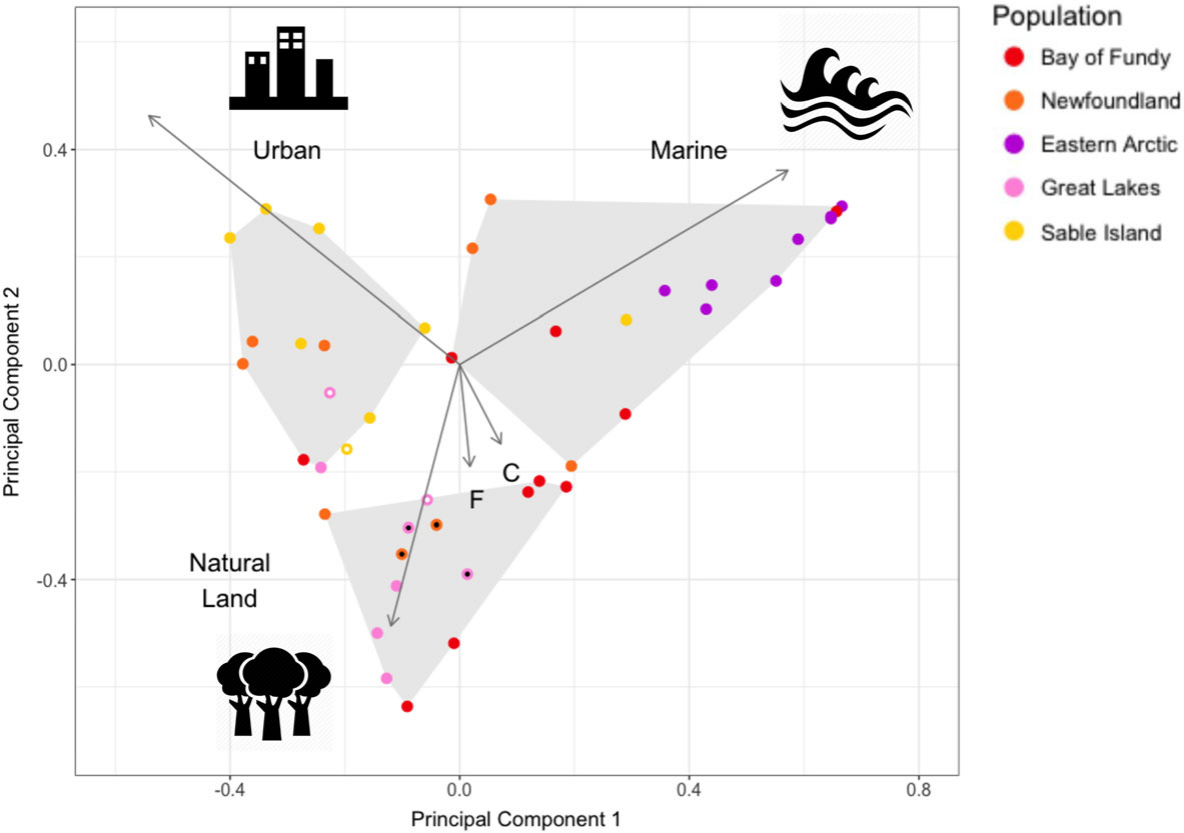
Individual values for the first two principal components from Principal Components Analysis of the proportion of time individual Herring Gulls spent in marine, natural land, cropland, urban, and freshwater habitats. The PCA loadings for each habitat type are indicated by arrows. Grey polygons enclose all individuals who spent the most time in marine, natural land, and urban habitats. Individuals who spent the most time in cropland and freshwater habitats are marked respectively with a black dot and white dot

Individual Herring Gulls predominantly used either marine, natural land or urban habitat, and individual time budgets in these habitat types differed by breeding population (Fig. 3). When we assessed individual habitat preferences using PCA, the first principal component explained 58% of the variance in habitat use, with loadings which contrasted the influence of marine (+ 0.72) and urban (− 0.68) habitats. The second principal component explained 27% of the variance, contrasting marine (+ 0.45) and urban (+ 0.58) from natural land (− 0.61) habitats. The three populations differed in their habitat use time budgets (PC1: *F* = 20.4, *p* < 0.001; PC2: *F* = 9.1, *p* < 0.001). Post-hoc Tukey tests showed that individuals from the eastern Arctic population used marine habitats more than individuals from the other populations (PC1). Individuals from the Great Lakes and the Bay of Fundy used natural land habitats more than individuals from the eastern Arctic and Sable Island (PC2). When looking at habitats where individuals spent the most time, all Herring Gulls from the eastern Arctic used marine habitats, 6 of 8 individuals from Sable Island used urban habitats, and individuals from the other three populations varied in which habitat type they used most (Fig. 3). Ten of eleven birds that specialized in urban habitats came from one of the Atlantic populations.

## Discussion

In this study, we compared the wintering distribution of Herring Gulls breeding in the eastern Arctic, the Great Lakes, Newfoundland, Sable Island, and the Bay of Fundy. Most individual Herring Gulls spent the winter within a home range of less than 20,000 km^2^. This is consistent with reports that individual Herring Gulls have high site fidelity between years [28] and often specialize in specific feeding locations [53]. Adult Herring Gulls in eastern North America appeared to exhibit strong migratory connectivity in this study, with populations consistently migrating to their own discrete wintering area. However, the winter home range of the Atlantic Canada breeding population overlaps with another resident population of Herring Gulls breeding on the Atlantic coast of the United States which were not tracked in this study [27]. In contrast, juvenile Herring Gulls banded as chicks in both the Atlantic and Great Lakes regions disperse more widely than adults, showing weaker migratory connectivity [27, 54].

All populations showed a preference for aquatic habitats. This is not surprising, as fish and intertidal organisms are typically the most common source of food for Herring Gulls [55–57]. Herring Gulls rely on open, unfrozen areas to access these food sources during the winter [27]. However, Herring Gulls from the eastern Arctic selected more strongly for marine habitats during the winter, whereas the other populations selected for freshwater habitats. In marine habitats, Herring Gulls from the Arctic were observed across the full extent of the continental shelf, while those from Atlantic Canada largely stayed close to shore. One environmental factor that may contribute differences between populations is the seasonality of these winter habitats. The winter home ranges of these populations differ in temperature, the extent of ice and snow, and the ecology of prey species (temperate freshwater vs temperate marine vs subtropical marine).

Although there was evidence that gulls from the Great Lakes, Bay of Fundy and Sable Island populations preferentially selected urban habitats, the greater availability of urban habitats in the two Atlantic populations means that their overall use of urban habitats is greater than the Great Lakes population. Virtually all individuals that spent more time in urban areas than other habitats came from one of the Atlantic populations. Interestingly, Robertson et al. [10] found the lowest survival in North American Herring Gulls was for Atlantic breeding birds, and they speculated that their likely higher use of urban or polluted habitats in the winter might be linked to lower survival. However, mechanisms by which habitat may negatively influence the survival of Herring Gulls during the winter are still unknown.

The differences we observed in habitat use between populations may not necessarily correspond to differences in diet. Although nesting habitat selection is correlated with the diet of Herring Gulls during the breeding season [24, 58], this same relationship has never been quantified for the wintering period. In Herring Gulls sampled from culls at a New York City airport, marine organisms were found in the stomachs of more than 60% of individual birds, and made up more than 50% of food volume [59]. It is possible Herring Gulls on the Atlantic coast may be able to forage efficiently in marine environments, potentially by taking advantage of fishing vessels [27], and thus the time in urban areas may reflect time spent loafing rather than foraging. Generally, the diet of Herring Gulls in New York City appears similar to diet of Herring Gulls in the Great Lakes and Atlantic Canada [56, 58–60], although there have been few studies of gull diets during the winter. Herring Gulls may not be actively selecting urban habitats, but may simply be returning to the same coastal habitats they inhabited before these habitats were altered by humans.

Another mechanism that may decrease survival of Herring Gulls in Atlantic Canada is direct mortality from human influences [54]. Herring Gulls are a common bycatch species in fisheries because they are attracted to vessels [61]. However, fisheries mortalities seem unlikely to be the source of significant differences in survival rates. Bycatch occurs in both the Gulf of Mexico and the Atlantic Region [62, 63], and reductions in fishing effort since 1992 have likely reduced the magnitude of seabird bycatch on the Atlantic Coast [64].

In urban and industrial areas, Herring Gulls may be harassed and shot as part of active management programs, particularly at airports [22]. For example, the large-scale culling of gulls appears to have played a significant role in the decline of Herring Gull populations across the UK during the last 45 years [65]. In eastern North America, the U.S. Fish and Wildlife Service has developed Potential Biological Removal models which suggest annual take of more than 16,725 (95% CI = 7788 – 25,397) Herring Gulls would lead to population declines in the region spanning Delaware Bay to Nova Scotia [66]. Between 2010 and 2013, the average annual take of Herring Gulls was 4445 in the northeastern United States [67]. However, more than 80% of gulls are lethally taken during the period from September to March [66]. Gull control programs in the United States may have a disproportionate effect on the Herring Gulls breeding in Atlantic Canada, which move into this region during this same period of the autumn and winter. To get a better sense of the impact of culling on gull populations in North America, it would be beneficial to conduct a review similar to Coulson’s [65] evaluation of the roles landfills and culling have played in the population dynamics of British Herring Gulls.

Herring Gulls have long been used as a model species for understanding contaminant dynamics [68], and understanding the nature of their full annual cycle and migratory connectivity could help us understand how contaminants are transferred geographically. The contaminant burden of Herring Gulls is known to be dependent, at least in part, on where birds migrate to and what they eat during the winter [69, 70]. Herring Gull eggs from the Great Lakes are known to have higher organochlorine burdens than eggs from Atlantic Canada and the Arctic [71]. These patterns could be attributed to specific environments for each population since they are geographically separated for their whole annual cycle. Interestingly, Gebbink et al. [72] found that perfluorinated compounds (flame retardants) were particularly high in Herring Gull eggs from Sable Island, which is consistent with their high degree of winter urban habitat use. Although gulls are typically regarded more as income breeders on the spectrum of endogenous-exogenous resource use [73, 74], factors like egg order, egg component, and environmental conditions influence the extent to which resources accumulated prior to the breeding season are deposited in eggs [75, 76].

## Conclusions

Our results provide new insights into winter habitat use by Herring Gulls in eastern North America. Herring Gulls breeding in the Eastern Arctic spent the clear majority of their time in marine environment of the Gulf of Mexico. Birds from the Great Lakes used a diverse set up habitats during the winter. While the three Atlantic populations also used a variety of habitats, the Newfoundland and Sable Island populations selected for urban habitats, and almost all individuals the specialized in urban habitats came from one of the three Atlantic populations. These conclusions provide are consistent with the hypothesis that lower survival rates for Herring Gulls in eastern North America may be related to characteristics of their wintering areas, suggesting it would be worthwhile to investigate both the foraging ecology and causes of direct mortality for Herring Gulls during the non-breeding season.

## Additional file

Additional file 1: Supplementary methods for state-space models and estimating home range bias. (DOCX 67 kb)
