# Correction to: Winter home range and habitat selection differs among breeding populations of herring gulls in eastern North America

**DOI:** 10.1186/s40462-019-0157-5

**Published:** 2019-04-12

**Authors:** Christine M. Anderson, H. Grant Gilchrist, Robert A. Ronconi, Katherine R. Shlepr, Daniel E. Clark, D. V. Chip Weseloh, Gregory J. Robertson, Mark L. Mallory

**Affiliations:** 1 Department of Biology, Acadia University, 33 Westwood Ave, Wolfville, NS B4P 2R6 Canada; 2 Wildlife Research Division, Environment and Climate Change Canada, National Wildlife Research Centre, Ottawa, ON K1S 5B6 Canada; 3 Canadian Wildlife Service, Environment and Climate Change Canada, 45 Alderney Dr, Dartmouth, NS B2Y 2N6 Canada; 4 Atlantic Lab for Avian Research, Department of Biology, University of New Brunswick, 10 Bailey Drive, P.O. Box 4400, Fredericton, NB E3B 5A3 Canada; 5 Massachusetts Department of Conservation and Recreation, Division of Water Supply Protection, 485 Ware Road, Belchertown, MA 01007 USA; 6 Canadian Wildlife Service, Environment and Climate Change Canada, 4905 Dufferin Ave, Toronto, ON M3H 5T4 Canada; 7 Wildlife Research Division, Environment and Climate Change Canada, 6 Bruce Street, Mount Pearl, NL A1N 4T3 Canada

**Correction to: Mov Ecol**

**https://doi.org/10.1186/s40462-019-0152-x**

Following publication of the original article [1], the authors reported that one of the authors’ names was spelled incorrectly. In this Correction the incorrect and correct author name are shown. The original publication of this article has been corrected.

Originally the author name was published as:

- Gregory J. Roberston

The correct author name is:

- Gregory J. Robertson
