# Supplementary material for: Winter home range and habitat selection differs among breeding populations of herring gulls in eastern North America
Source: Mov Ecol. 2019 Mar 7;7:8. doi: 10.1186/s40462-019-0152-x (PMC6404351; doi:10.1186/s40462-019-0152-x)

*State Space Models*

Tracking data (Argos Doppler data in particular) were recorded at irregular time intervals, and are known often to be less precise than the location error estimates provided by the manufacturer (Vincent et al. 2002). The data we collected from different sites varied greatly in their sampling frequency, which in turn can strongly influence the interpretation of movement metrics such as distance and directness (Tanferna et al. 2012). To compensate for these issues, we used Bayesian hierarchical switching state-space models to estimate locations at regular 24 h intervals (Jonsen et al. 2013, 2015). State-space models estimate the most probable movement path of an individual using two components. First, the process model describes the movement path of an individual as a first-difference correlated random walk, switching between two data-driven behavioural states (travelling and foraging) that dictate the distributions of speed and turning angles between locations. Second, the observation model relates the observed data points to the animal’s unobserved location from the process model. The observation model characterizes measurement error by using independently verified data from Vincent et al. (2002) to determine the distribution of each ARGOS location error class. Fitting all individuals within a population using the same state-space model improves the accuracy of location estimates. Additional details about the general parameterization of these models are described in Jonsen et al. (2005).

Prior to modelling, we removed duplicate locations and applied a speed filter of 200 km/hr to remove outlier locations from each dataset, enabling more accurate estimates (Freitas et al. 2008; Freitas 2012). Based on run length diagnostics of model test-runs (Raftery and Lewis 1991), we fit state-space models to the dataset using two chains of 400,000 Monte Carlo Markov Chain (MCMC) samples. We discarded the first 50,000 samples as a burn-in, and retained only every 50th sample of the remaining 350,000 samples to reduce autocorrelation. Using the R package CODA (Plummer et al. 2006), we checked the parameter estimates from the remaining 7000 samples for convergence by examining: (1) trace plots of model parameters for good mixing and stationary chains, (2) autocorrelation plots for independence between locations, and (3) density plots, Gelman and Rubin (1992) diagnostics, and Geweke (Geweke 1992) diagnostics for evidence that posterior distributions were unimodal. We also visually compared the modeled locations to the observed locations. We removed locations that were modeled beyond one day of an observed location point, as the state-space model tended to provide biologically unrealistic estimates during large data gaps.

Figure S1: Effects of sample size (number of tracked birds) on the calculated 90% kernel utilization distributions.


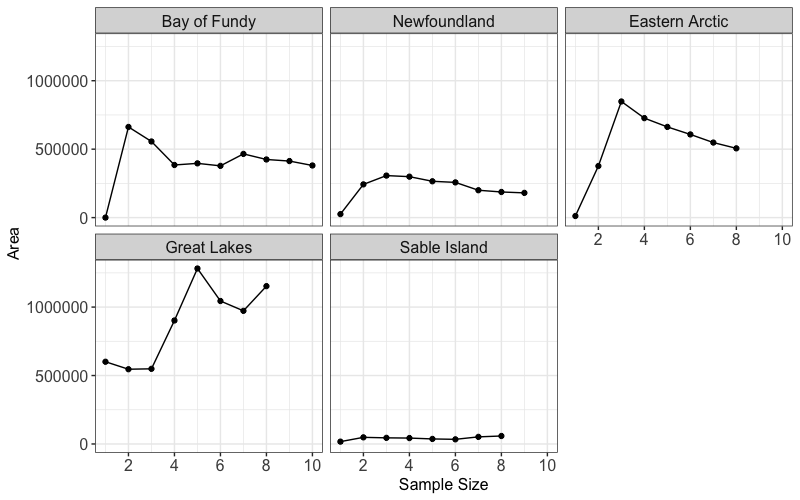

Supplement: Supplementary file 1 — Supplementary methods for state-space models and estimating home range bias. (DOCX 67 kb) [file 40462_2019_152_MOESM1_ESM.docx]
